# Sphingosine kinase 1 mediates diabetic renal fibrosis via NF-κB signaling pathway: involvement of CK2α

**DOI:** 10.18632/oncotarget.21640

**Published:** 2017-10-04

**Authors:** Junying Huang, Jingyan Li, Zhiquan Chen, Jie Li, Qiuhong Chen, Wenyan Gong, Peiqing Liu, Heqing Huang

**Affiliations:** ^1^ Laboratory of Pharmacology & Toxicology, School of Pharmaceutical Sciences, Sun Yat-sen University, Guangzhou 510006, China; ^2^ College of Life Sciences, Guangzhou University, Guangzhou 510006, China; ^3^ National and Local United Engineering Lab of Druggability and New Drugs Evaluation, Guangzhou 51000, China

**Keywords:** sphingosine kinase 1, casein kinase 2α subunit, diabetic nephropathy, NF-κB pathway, renal fibrosis

## Abstract

Sphingosine kinase 1 (SphK1) plays a pivotal role in regulating diabetic renal fibrotic factors such as fibronectin (FN) and intercellular adhesion molecule-1 (ICAM-1). Especially, activation of SphK1 is closely linked to the body inflammatory reaction. Casein kinase 2α subunit (CK2α), a protein kinase related to inflammatory reaction, influences diabetic renal fibrosis and expressions of FN and ICAM-1 via NF-κB pathway. However, the mechanism by which SphK1 mediates diabetic renal fibrosis has not yet fully elucidated. The current study is aimed to investigate if SphK1 mediates diabetic renal fibrotic pathological process via inflammatory pathway and activation of CK2α. The following findings were observed: (1) Expressions of SphK1 were upregulated in kidneys of diabetic mice and rats; (2) Knockdown of SphK1 expression suppressed high glucose (HG)-induced NF-κB nuclear translocation and expressions of FN and ICAM-1; (3) Compared with C57 diabetic mice, SphK1^-/-^ diabetic mice exhibited less renal fibrotic lesions, FN accumulation and NF-κB nuclear accumulation in glomeruli of kidneys; (4) SphK1 mediated phosphorylation of CK2α, while CK2α knockdown depressed SphK1-induced activation of NF-κB pathway. This study indicates the essential role of SphK1 in regulating activation of CK2α and diabetic renal fibrotic pathological process via NF-κB.

## INTRODUCTION

Diabetic nephropathy (DN), also known as diabetic glomerulosclerosis characterized by renal fibrosis, is a major chronic microvascular complication of diabetes mellitus (DM) and is the leading cause of morbidity and mortality in diabetic patients with end-stage renal failure [1, 2]. Accumulating evidence suggests that the pathological mechanism of diabetic renal fibrosis is complicated and closely related to numerous factors. Nonenzymatic glycation of proteins, glucolipid metabolism disorders, inflammation, oxidative stress, polyol pathway and MAPK signaling pathway are believed to be implicated in the development of diabetic renal fibrosis [3, 4]. However, the pathological mechanism of DN has not been fully elucidated. Increasing number of studies suggest that inflammation is involved in the development and progression of DN, and it even receives serious attention that diabetic renal fibrotic disease is one kind of renal chronic inflammatory response [5–7]. NF-κB, a classic inflammatory signaling pathway, regulates expressions of numerous genes regarding inflammatory response in the process of clinical and experimental kidney disease [8]. Five proteins have been found in the mammalian NF-κB family: RelA (p65), RelB, c-Rel, p50/p105 and p52/p100 [9]. Under non-diabetic state, IκB acts as an anchor protein to combine with NF-κB in the cytoplasm, forming an inactive complex to inhibit the activation of NF-κB signaling pathway. In response to stimulation, IκB kinase (IKK) is phosphorylated and then phosphorylates IκB to cause ubiquitin-mediated degradation of IκB via proteasome, promoting release of NF-κB from inactive complex and then allowing NF-κB to translocate from cytoplasm into nucleus to trigger target genes expression [10, 11]. Therefore, further study about the molecular mechanism of diabetic renal fibrosis and inflammation is imperative for preventing the progression of diabetic renal failure and exploring an approach to DN treatment.

Protein kinase CK2 (formerly casein kinase II), a highly conserved and ubiquitous second messenger-independent serine/threonine protein kinase, is widely distributed in the cytoplasm and nucleus of eukaryotic cells and phosphorylates more than 300 substrates to influence a vast array of biological and pathological processes [12]. CK2α, a catalytic subunit of CK2, has a significant serine/threonine kinase activity and even been considered to be significant in numerous key biological processes such as cell proliferation, differentiation and apoptosis [13–16]. What’s more, CK2α participates in development and progression of diseases including angiogenesis, glomerulonephritis, organogenesis and cancer [17–20]. Our previous study shows that activated CK2α provokes inflammatory nuclear factor NF-κB through interacting with IκB to increase its degradation, upregulating expressions of inflammatory fibrotic factors including FN and ICAM-1 under diabetic state [21].

In recent years, the relationship between SphK1 and diabetic renal fibrosis also receives serious attention. SphK1 is activated under stimulation of advanced glycation end products (AGEs) or oxidative stress during the period of diabetes, accompanied by formation of S1P to mediate the biological and physiological activities of cells [22, 23]. Several studies demonstrate that sphingosine-1-phosphate (S1P), a lipid involved in cellular proliferation, is a phosphorylated product of sphingosine catalyzed by SphK1 and promotes cell proliferation of GMCs [24]. Moreover, activity of SphK1 and level of S1P are even upregulated in AGEs-cultured GMCs [25]. *In vivo*, compared with control groups, STZ-induced diabetic rats show higher activity of SphK1 and level of S1P in kidneys [26].

However, the mechanism by which SphK1 mediates diabetic renal fibrosis has not yet fully elucidated. It is reported that activity and protein level of SphK1 are upregulated in inflammation-related diseases [27–30]. Various inflammatory signals including interleukin1β (IL-1β), interferon γ (IF-γ) and lipopolysaccharide (LPS) promote SphK1 expression, indicating the underlying regulatory role of SphK1 on inflammatory reaction [31, 32]. It is also provided that renal inflammatory response in the context of DN is one of the important hallmarks of diabetic renal fibrosis, and CK2α as well as SphK1 performs as a regulator in process of DN. In view of these, it catches our interests whether SphK1-mediated diabetic renal pathological process via inflammatory pathway is dependent on activation of CK2α. In this study, we assessed the regulatory function of SphK1 on activation of NF-κB and CK2α, and tried to explore the relationship between SphK1 and CK2α in HG-induced GMCs and STZ-induced SphK1^-/-^ diabetic mice.

## RESULTS

### The expressions of SphK1 were upregulated in kidneys of diabetic animals

**Figure 1:**
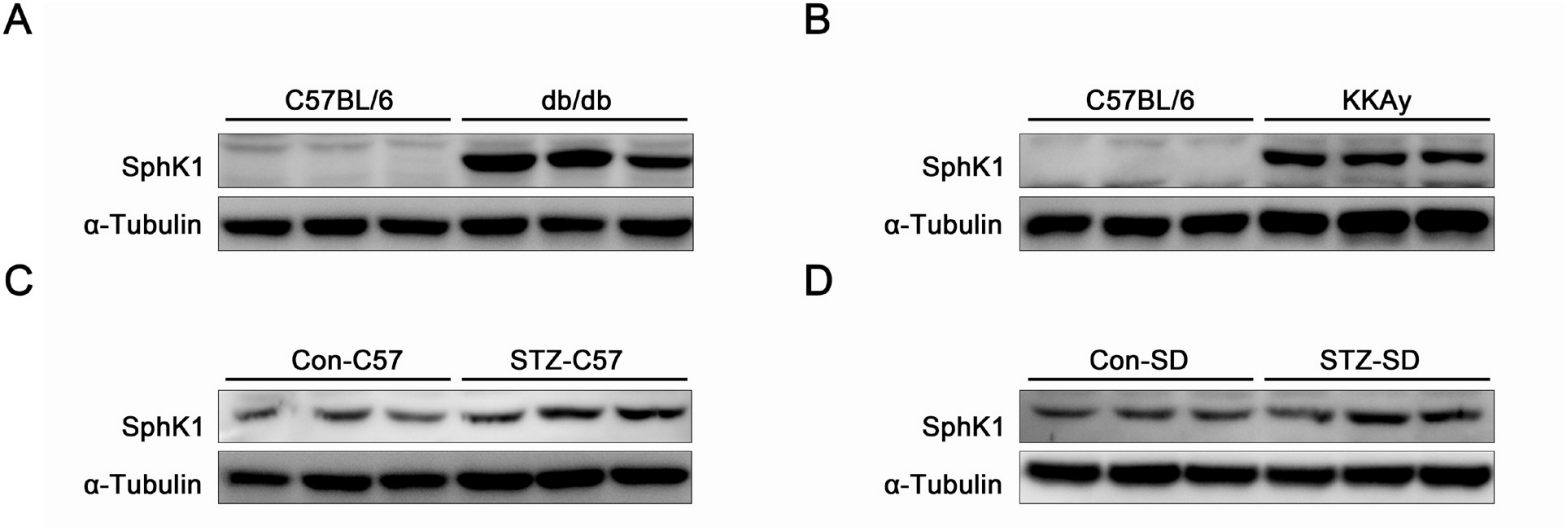
The expressions of SphK1 were upregulated in kidneys of diabetic animals **(A-D)** The expressions of SphK1 in kidneys of db/db diabetic mice, KKAy diabetic mice, STZ-induced C57 diabetic mice and STZ-induced SD rats were detected by western blotting (*n* = 3 different animals).

Protein expressions of SphK1 were examined in kidneys of diabetic animals by western blotting. Compared with normal C57/BL6 mice, expressions of SphK1 were visibly upregulated in kidneys of spontaneous diabetic animals including db/db diabetic mice and KKAy diabetic mice (Figure 1A & 1B). Data further indicated that increases of SphK1 expression occurred in kidneys of STZ-induced C57/BL6 diabetic mice and SD diabetic rats by comparing with the control animals (Figure 1C & 1D). The above results described here showed an essential link between SphK1 and DN.

### The activity and protein level of SphK1 were upregulated in high glucose-induced GMCs

**Figure 2:**
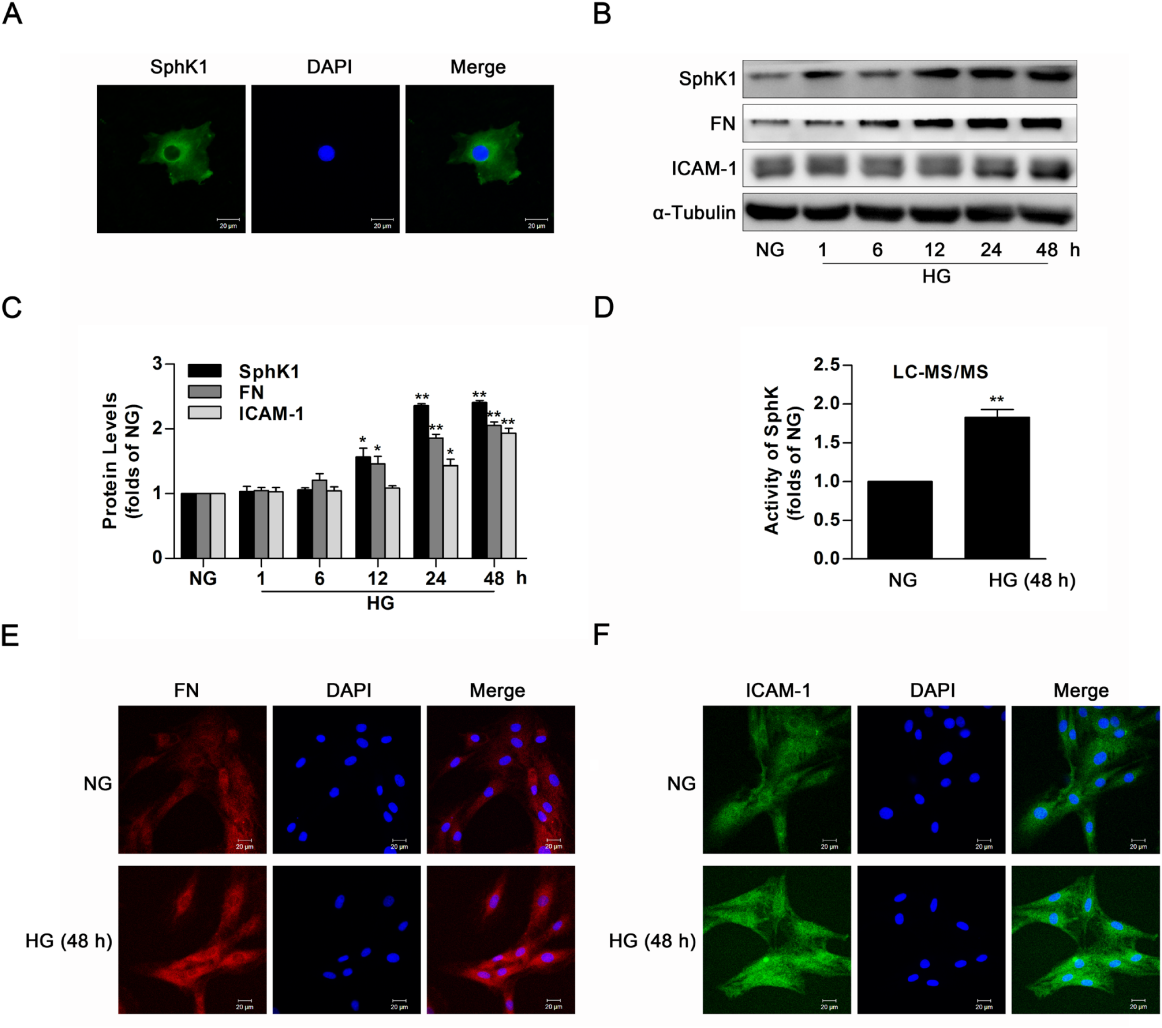
The activity and protein level of SphK1 were upregulated in high glucose-induced GMCs **(A)** Immunofluorescent stain showed the subcellular distribution of SphK1 in NG-cultured GMCs. Green and blue stain respectively indicate SphK1 and nuclei. **(B** and **C)** Expressions of SphK1, FN and ICAM-1 were upregulated in 30 mM HG-induced GMCs in a time-dependent manner (NG, 1, 6, 12, 24, 48 h). Values represent means ± S.E. (*n* = 3 independent experiments. *P < 0.05, **P < 0.01 vs. NG by one-way ANOVA). **(D)** Activity of SphK was upregulated after treatment of high glucose for 48 h by LC-MS/MS. Values represent means ± S.E. (*n* = 3 independent experiments.**P < 0.01 vs. NG by Student’s t test). **(E** and **F)** Fluorescent levels of FN and ICAM-1 improved by immunofluorescent stain. Blue, red and green stain respectively indicate nuclei, FN and ICAM-1.

Data of immunofluorescence showed evident expression of SphK1 in the cytoplasm of GMCs (Figure 2A). To investigate the activation of SphK1 in GMCs during diabetes, activity and protein level of SphK1 were measured by LC-MS/MS and western blotting respectively. During continuous HG stimulation, protein level of SphK1 as well as renal fibrotic factors containing FN and ICAM-1 was upregulated in a time-dependent manner, reaching a peak value at 48 h (Figure 2B & 2C). Similarly, activity of SphK was upregulated (Figure 2D), and fluorescent levels of FN and ICAM-1 improved after HG treatment for 48 h (Figure 2E & 2F).

### Overexpression of SphK1 elevated expressions of FN and ICAM-1 in normal glucose-cultured GMCs

**Figure 3:**
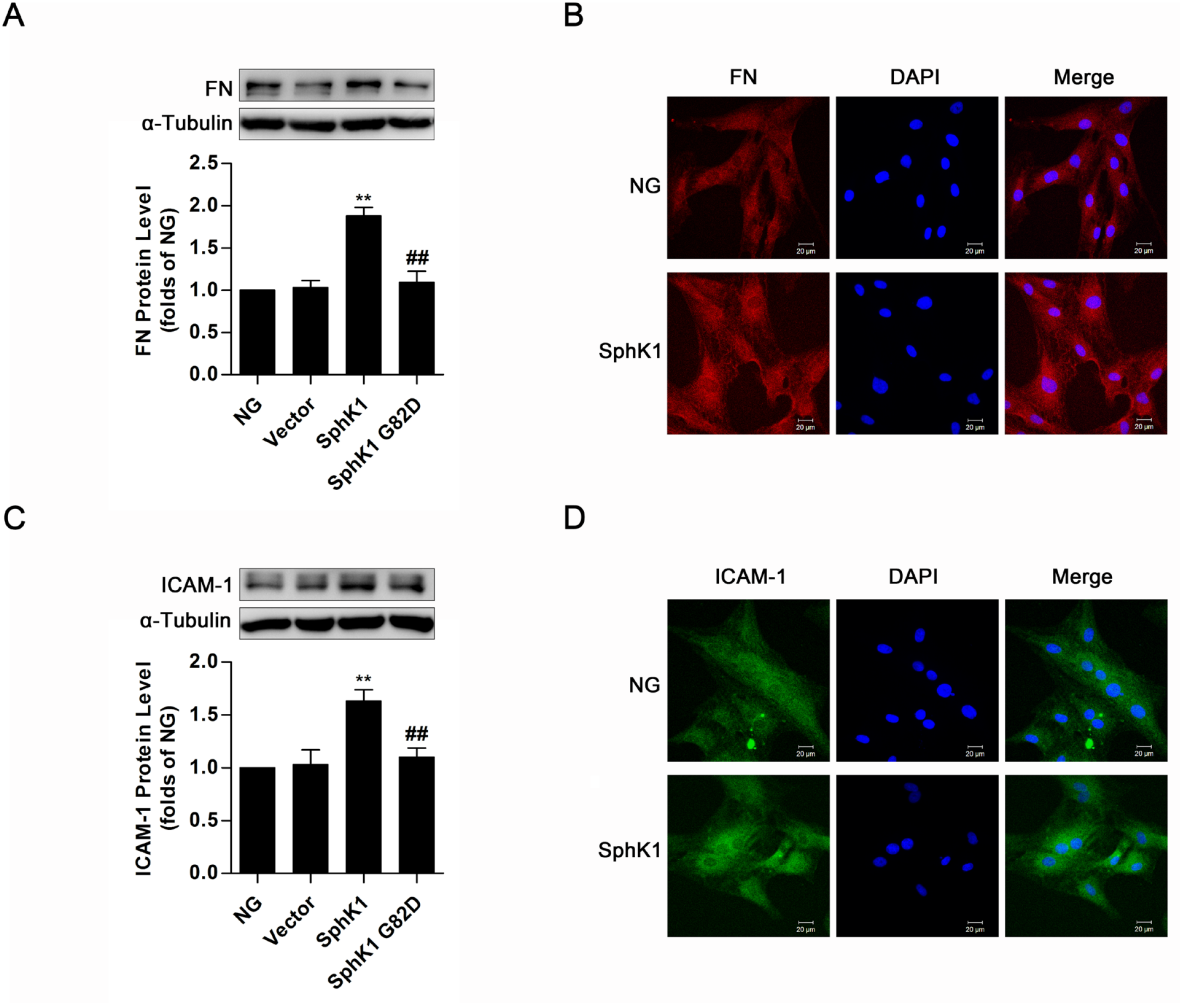
Overexpression of SphK1 elevated expressions of FN and ICAM-1 in normal glucose-cultured GMCs **(A** and **C)** Data of western blotting showed that overexpression of wild-type SphK1, but not the dominant negative mutant SphK1 G82D, obviously increased expressions of FN and ICAM-1. Values represent means ± S.E. (*n* = 3 independent experiments. **P < 0.01 vs. NG; ^##^P < 0.01 vs. SphK1 by one-way ANOVA). **(B** and **D)** Fluorescent levels of FN and ICAM-1 improved by immunofluorescent stain after overexpression of wild-type SphK1. Blue, red and green stain respectively indicate nuclei, FN and ICAM-1.

For investigating the effect of SphK1 on inflammatory fibrotic factors FN and ICAM-1 further, plasmids of SphK1 G82D, a dominant negative SphK1 mutant, and wild-type SphK1 were overexpressed in normal glucose (NG)-cultured GMCs. Compared with the normal control, significant increases of FN and ICAM-1 occurred in GMCs expressing wild-type SphK1 but not in cells expressing SphK1 G82D (Figure 3A & 3C). Consistently, FN and ICAM-1 fluorescent levels improved in SphK1-overexpressed GMCs (Figure 3B & 3D).

### Knockdown of SphK1 decreased expressions of FN and ICAM-1 in normal glucose-cultured GMCs

**Figure 4:**
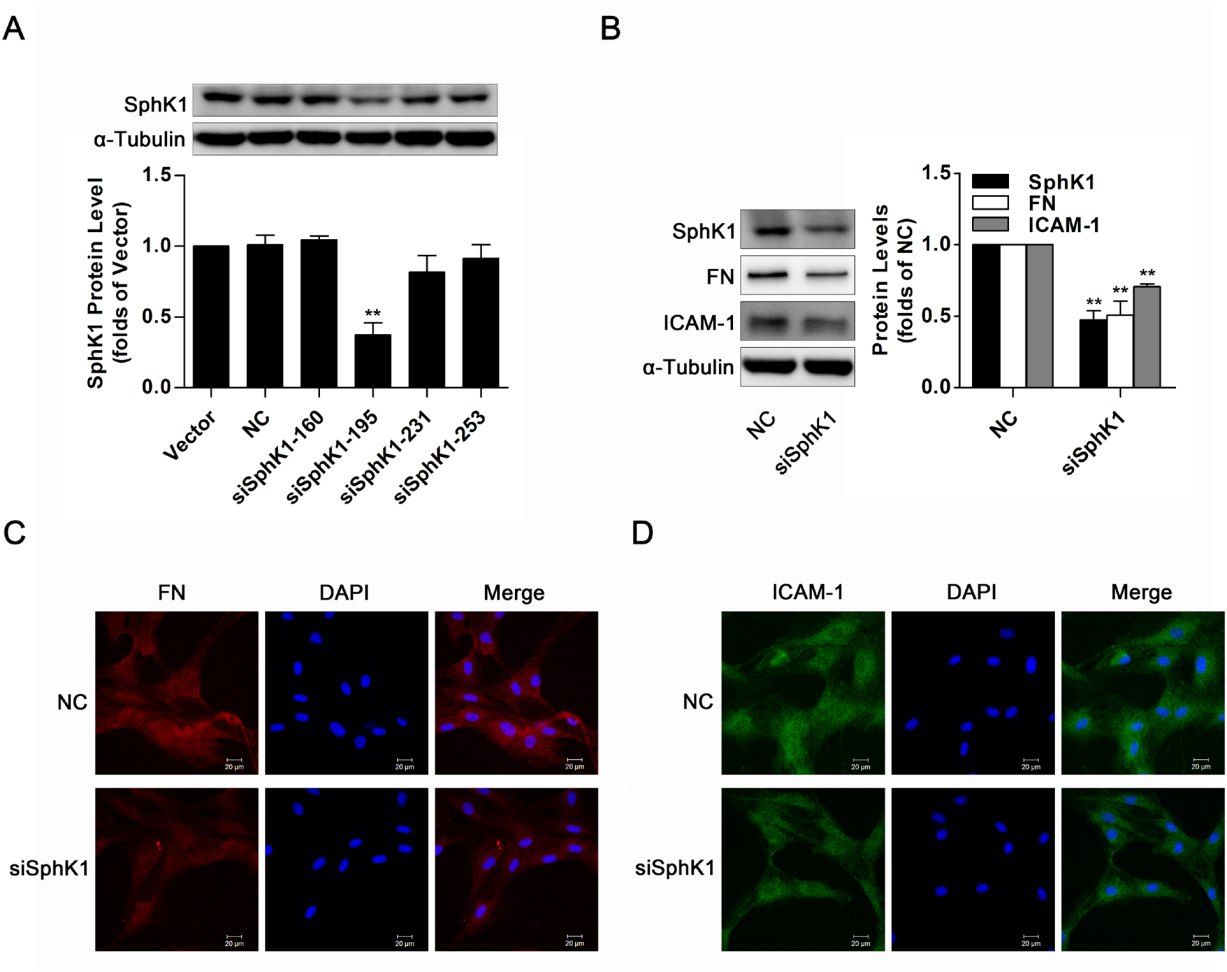
Knockdown of SphK1 decreased expressions of FN and ICAM-1 in normal glucose- cultured GMCs **(A)** Knockdown of SphK1 expression was detected by western blotting. GMCs were transfected with negative control or four pairs of siRNA oligonucleotides targeting SphK1 and then cultured for 48 h. Values represent means ± S.E. (*n* = 3 independent experiments. **P < 0.01 vs. Vector by one-way ANOVA). **(B-D)** Data from western blotting and immunofluorescent stain showed that knockdown of SphK1 reduced expressions of FN and ICAM-1 in GMCs. Blue, red and green stain respectively indicate nuclei, FN and ICAM-1. Values represent means ± S.E. (*n* = 3 independent experiments. **P < 0.01 vs. NC by Student’s t test).

The aforementioned data suggested overexpression of wild-type SphK1 abundantly augmented FN and ICAM-1 protein levels, and we further measured expressions of FN and ICAM-1 in SphK1-silenced GMCs. Knockdown of SphK1 was in GMCs by the method of siRNA (Figure 4A), accompanied by decreases of FN and ICAM-1 in NG-cultured GMCs (Figure 4B). Moreover, knockdown of SphK1 reduced FN and ICAM-1 fluorescent levels in NG-cultured GMCs (Figure 4C & 4D).

### Overexpression of SphK1 elevated nuclear translocation of NF-κB in normal glucose-cultured GMCs

**Figure 5:**
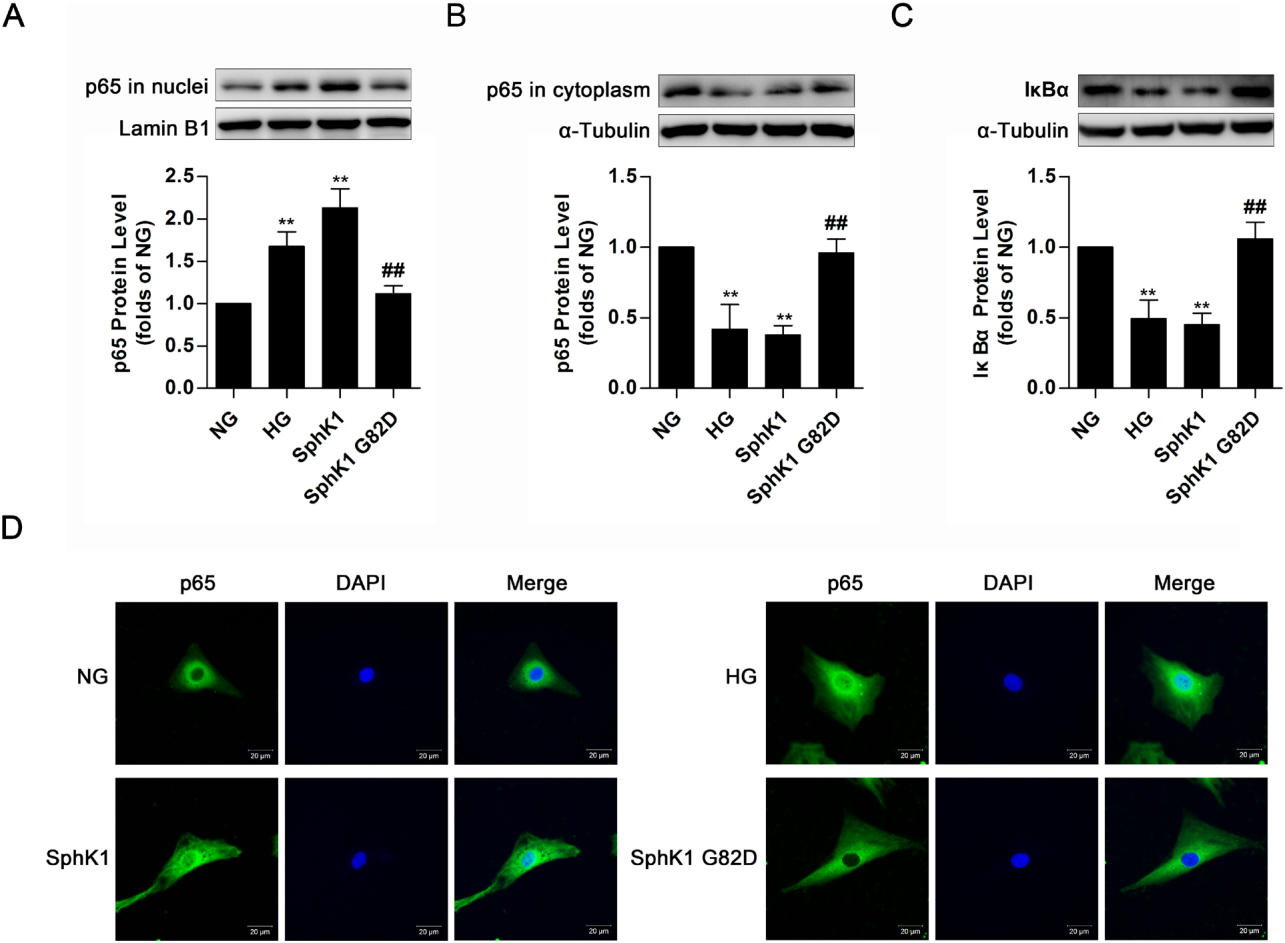
Overexpression of SphK1 elevated nuclear translocation of NF-κB in normal glucose-cultured GMCs **(A-C)** Overexpression of SphK1 as well as HG treatment, but not SphK1 G82D, augmented nuclear NF-κB p65 protein level and decreased cytoplasmic NF-κB p65 and IκBα protein levels. Values represent means ± S.E. (*n* = 3 independent experiments. **P < 0.01 vs. NG; ^##^P < 0.01 vs. SphK1 by one-way ANOVA). **(D)** Overexpression of SphK1 increased fluorescent level of NF-κB p65 in nucleus by immunofluorescent stain. Blue and green stain respectively indicate nuclei and NF-κB p65.

Upon HG stimulation for 30 min, IκB dissociates from NF-κB to be degraded via proteasome, triggering nuclear translocation of free NF-κB to boost FN and ICAM-1 expressions in GMCs [11]. Since the above data revealed that activated SphK1 was implicated in expressions of FN and ICAM-1, we further examined activation of NF-κB in GMCs expressing wild-type SphK1 or SphK1 G82D. Overexpression of SphK1, as well as HG stimulation for 30 min, improved nuclear NF-κB p65 protein level and decreased protein levels of cytoplasmic NF-κB p65 and IκBα, whereas SphK1 G82D, had no obvious effect on NF-κB nuclear translocation (Figure 5A-5C). Immunofluorescence assay was performed to illustrate that nuclear NF-κB p65 fluorescent level improved under HG stimulus or SphK1 overexpression (Figure 5D).

### Knockdown of SphK1 decreased nuclear translocation of NF-κB in high glucose-induced GMCs

**Figure 6:**
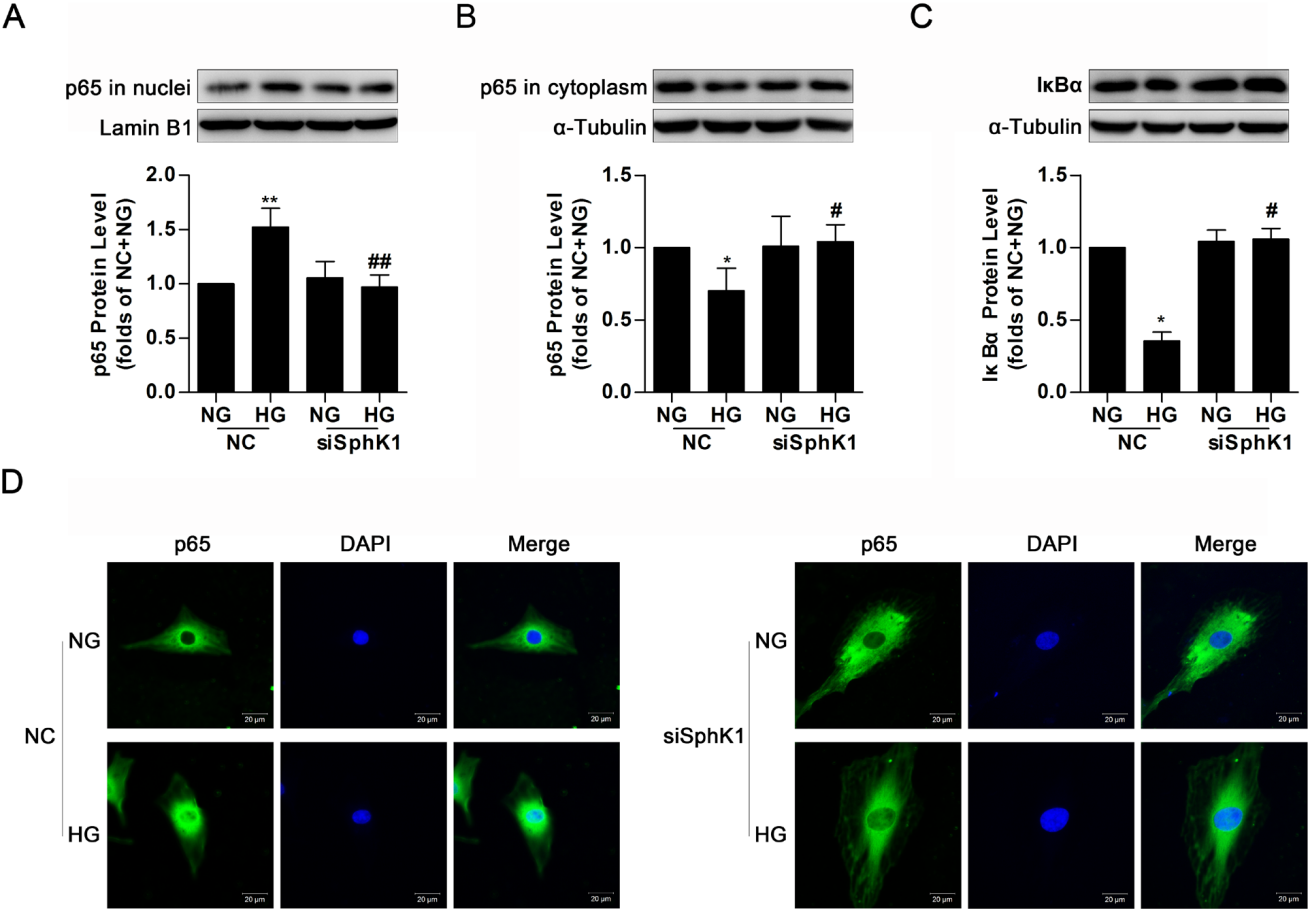
Knockdown of SphK1 decreased nuclear translocation of NF-κB in high glucose-induced GMCs **(A-C)** HG stimulus increased nuclear NF-κB p65, decreased cytoplasmic NF-κB p65 and IκBα, but all of these changes were completely reversed by knockdown of SphK1. Values represent means ± S.E. (*n* = 3 independent experiments. *P < 0.05, **P < 0.01 vs. NC+NG; ^#^P < 0.05, ^##^P < 0.01 vs. NC+HG by one-way ANOVA). **(D)** Impact of SphK1 knockdown on fluorescent level of NF-κB p65 were showed by immunofluorescent stain. Blue and green stain respectively indicate nuclei and NF-κB p65.

By the above experiment results, it was demonstrated that either SphK1 overexpression or HG stimulus provoked nuclear translocation of NF-κB in GMCs. Simultaneously, knockdown of SphK1 even lowered nuclear NF-κB p65 protein level, increased cytoplasmic NF-κB p65 protein level and occluded degradation of IκBα in HG-treated GMCs (Figure 6A-6C). Immunofluorescence assay was also carried out to illustrate that knockdown of SphK1 reduced HG-induced nuclear translocation of NF-κB in GMCs (Figure 6D).

### SphK1^-/-^ diabetic mice exhibited less renal fibrotic lesions, FN accumulation and NF-κB nuclear accumulation in glomeruli of kidneys

**Figure 7:**
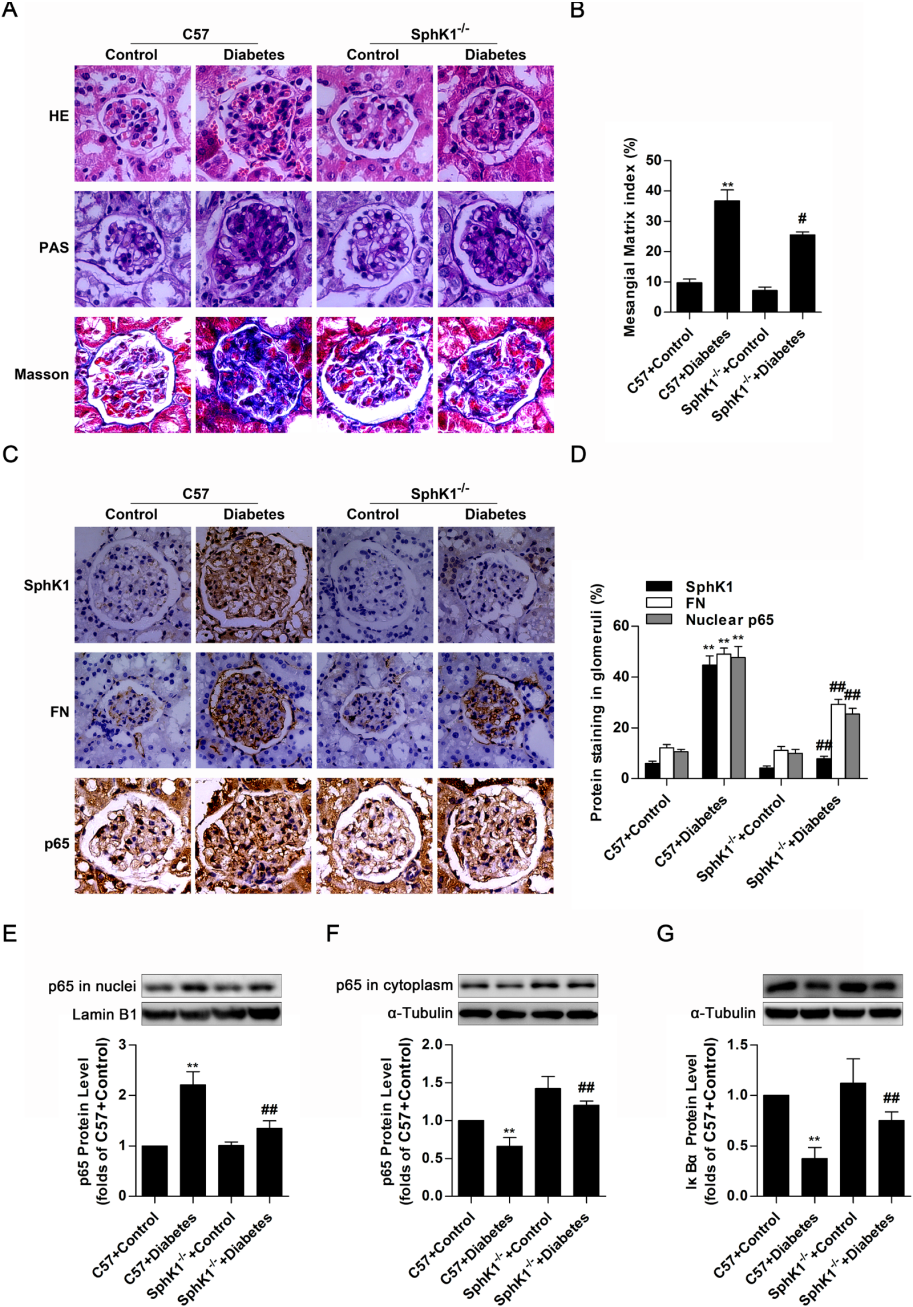
SphK1^-/-^ diabetic mice exhibited less renal fibrotic lesions, FN accumulation and NF-κB nuclear accumulation in glomeruli of kidneys **(A)** Glomerular histopathology analysis of HE, PAS and Masson stain (400 × magnification) showed the renal lesions. Pictures displayed representative glomeruli in kidneys of mice. Green in Masson stain indicates collagenous fibers. **(B)** The pathological analysis of glomerular was performed using PAS stain (400 × magnification). Values represent means ± S.E. (*n* = 3 different mice. **P < 0.01 vs. C57+Control; ^#^P < 0.05 vs. C57+Diabetes by one-way ANOVA). **(C** and **D)** Immunohistochemical stain showed the expressions of SphK1, FN and nuclear NF-κB p65 in paraffin-embedded kidney tissues (400 × magnification). Values represent means ± S.E. (*n* = 3 different mice. **P < 0.01 vs. C57+Control; ^##^P < 0.01 vs. C57+Diabetes by one-way ANOVA). **(E-G)** Detached nuclear and cytoplasmic extracts from kidneys according to nuclear extract kit were subjected to western blotting for detection of nuclear NF-κB p65, cytoplasmic NF-κB p65 and IκBα. Values represent means ± S.E. (*n* = 3 different mice. **P < 0.01 vs. C57+Control; ^##^P < 0.01 vs. C57+Diabetes by one-way ANOVA).

The *in vitro* experiments described here demonstrated that SphK1 exerted regulatory effects on nuclear transcriptional factor NF-κB as well as inflammatory fibrotic factors containing FN and ICAM-1 in HG-induced GMCs. However, further work *in vivo* is required for elucidating the essential role of SphK1 in diabetic animals. Glomerular lesions in kidneys of STZ-induced C57 diabetic mice and SphK1^-/-^ diabetic mice were detected, and data of hematoxylin-eosin (HE) stain and periodic acid-schiff (PAS) stain suggested that SphK1^-/-^ diabetic mice exhibited less renal lesions such as extracellular matrix overexpression, mesangial area expansion, basement membrane thickening and adhesion arising between glomerulus and renal capsule than C57 diabetic mice, followed by reduced mesangial matrix index. Masson stain further indicated that decrease of collagenous fibers generation occurred in SphK1^-/-^ diabetic mice by comparing with C57 diabetic mice (Figure 7A & 7B). Except for decrease of glomerular structural lesions, SphK1^-/-^ diabetic mice displayed less FN accumulation and NF-κB p65 nuclear accumulation in glomeruli of kidneys versus C57 diabetic mice (Figure 7C & 7D). Another detection by western blotting even showed that NF-κB p65 protein level increased in nuclear extract of kidneys, while cytoplasmic NF-κB p65 as well as IκBα decreased in kidneys of C57 diabetic mice. Remarkably, SphK1^-/-^ diabetic mice showed less STZ-induced increase of nuclear NF-κB p65 and decrease of cytoplasmic NF-κB p65 as well as IκBα than C57 diabetic mice (Figure 7E-7G). The above results pointed out a close correlation between SphK1 and DN, as well as activation of NF-κB.

### Knockdown of CK2α decreased nuclear translocation, transcriptional activity and DNA binding activity of NF-κB in SphK1-overexpressed GMCs

**Figure 8:**
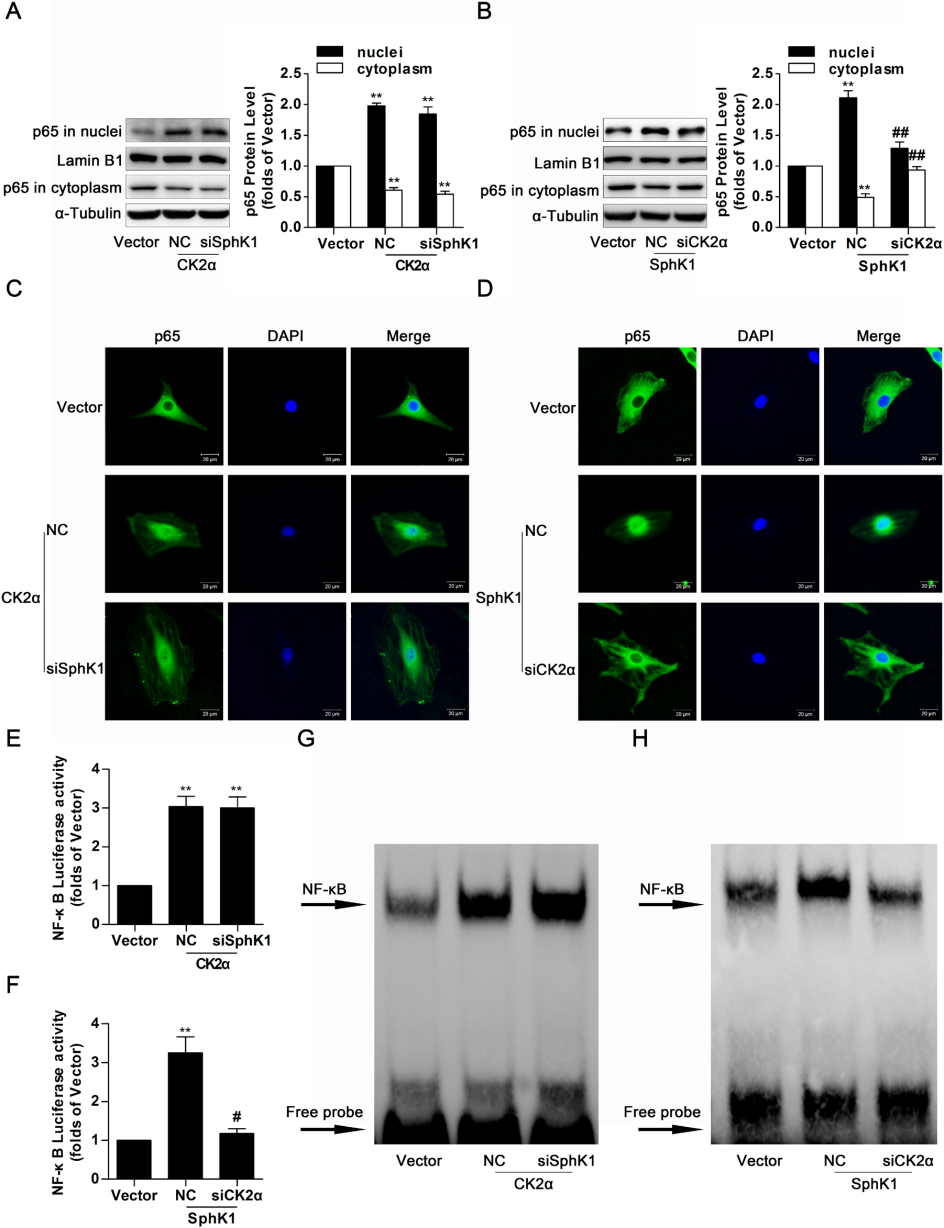
Knockdown of CK2α decreased nuclear translocation, transcriptional activity and DNA binding activity of NF-κB in SphK1-overexpressed GMCs **(A** and **C)** Data of western blotting and immunofluorescence showed that knockdown of SphK1 caused no evident change on nuclear translocation of NF-κB p65 in CK2α-overexpressed GMCs. Values represent means ± S.E. (*n* = 3 independent experiments. **P < 0.01 vs. Vector by one-way ANOVA). **(B** and **D)** However, nuclear NF-κB p65 was extremely downregulated and cytoplasmic NF-κB p65 was improved after CK2α knockdown in SphK1-overexpressed GMCs. Values represent means ± S.E. (*n* = 3 independent experiments. **P < 0.01 vs. Vector; ^##^P < 0.01 vs. SphK1+NC by one-way ANOVA). **(E-H)** Transcriptional activity and DNA binding activity of NF-κB were effectively inhibited after CK2α knockdown in SphK1-overexpressed GMCs, while knockdown of SphK1 showed no effective change on transcriptional activity and DNA binding activity of NF-κB in CK2α-overexpressed GMCs. Values represent means ± S.E. (*n* = 3 independent experiments. **P < 0.01 vs. Vector; ^#^P < 0.05 vs. SphK1+NC by one-way ANOVA).

Previous work and the above experiments established that SphK1 and CK2α performed as important regulators on FN and ICΑM-1expressions, as well as activation of NF-κB, suggesting that SphK1 and CK2α were jointly implicated in the NF-κB-mediated process of diabetic renal fibrosis. However, it remains unknown if there is a close interaction between SphK1 and CK2α, and what are associated to the regulatory mechanism. Results of western blotting and immunofluorescence indicated that knockdown of SphK1 caused no marked change on nuclear translocation of NF-κB p65 in CK2α-overexpressed GMCs (Figure 8A & 8C), whereas nuclear NF-κB p65 was extremely downregulated and cytoplasmic NF-κB p65 was improved after CK2α knockdown in SphK1-overexpressed GMCs (Figure 8B & 8D).

It was pointed out that CK2α may be the downstream target protein of SphK1, and nuclear translocation of NF-κB was mediated by SphK1 via CK2α. We further investigated the role of CK2α on transcription activity and DNA binding activity of NF-κB in SphK1-induced GMCs by the methods of dual luciferase reporter assay and EMSA assay. Data described here indicated that knockdown of SphK1 failed to suppress transcriptional activity and DNA binding activity of NF-κB in CK2α-overexpressed GMCs (Figure 8E & 8G), but activation of NF-κB were effectively inhibited after CK2α knockdown in SphK1-overexpressed GMCs (Figure 8F & 8H).

### SphK1 mediated the activation of CK2α in GMCs

**Figure 9:**
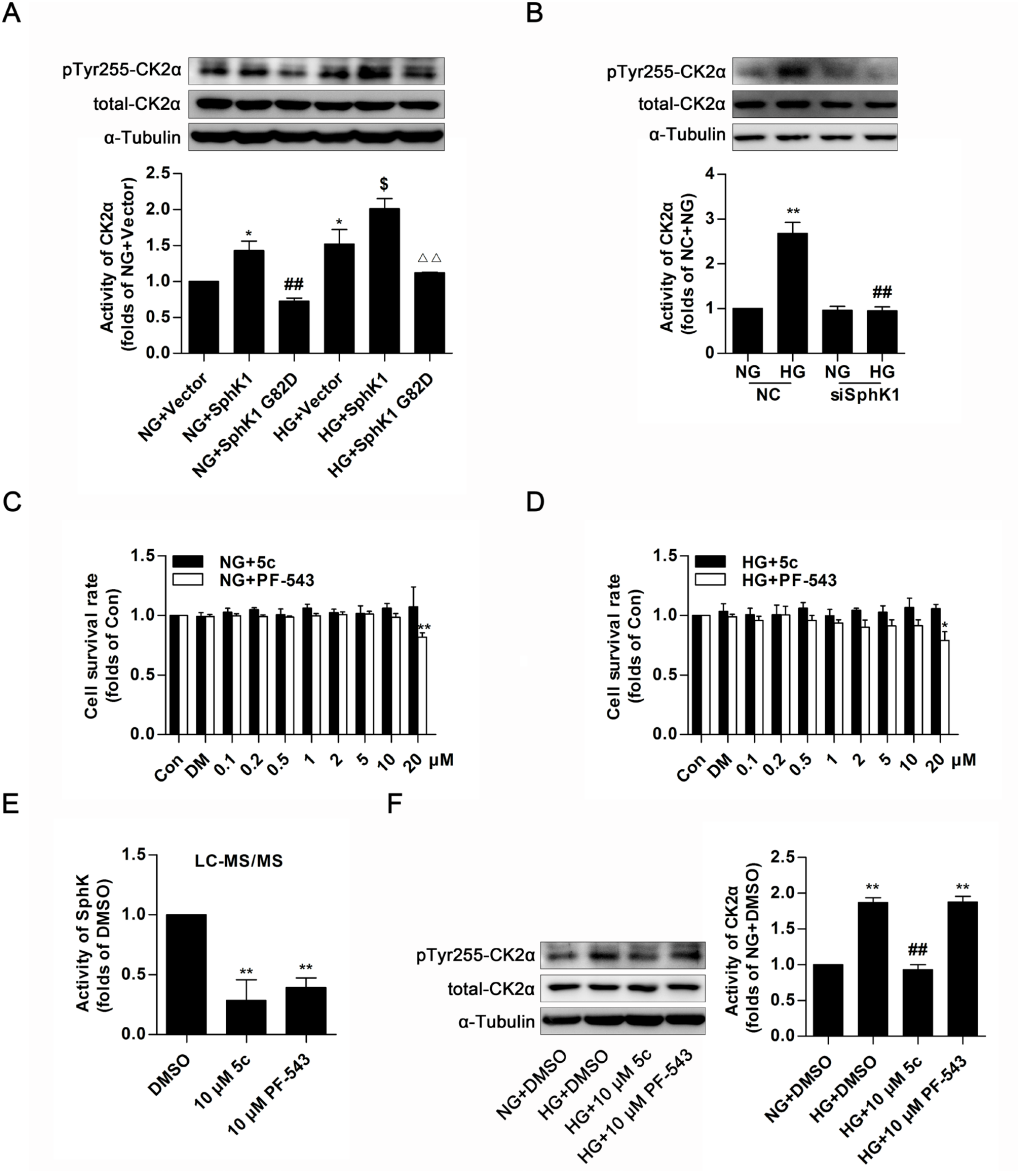
SphK1 mediated the activation of CK2α in GMCs **(A** and **B)** Protein level of pTyr255 CK2α was upregulated after SphK1 overexpression in diabetic state or in non-diabetic state, while knockdown of SphK1 suppressed protein level of pTyr255 CK2α in HG-induced GMCs. Values represent means ± S.E. (*n* = 3 independent experiments. *P < 0.05 vs. NG+Vector;^##^P < 0.01 vs. NG+SphK1;^$^P < 0.05 vs. HG+ Vector; ^∆∆^P < 0.01 vs. HG+SphK1 by one-way ANOVA.**P < 0.01 vs. NC+NG;^##^P < 0.01 vs. NC+HG by one-way ANOVA). **(C** and **D)** MTT assay indicated effects of PF-543 or 5c on cell proliferation in a dose-dependent manner, and no obvious inhibitory effects were caused when their concentrations was lower than 20 μM in NG or HG-cultured GMCs. Values represent means ± S.E. (*n* = 3 independent experiments. *P < 0.05, **P < 0.01 vs. Con by one-way ANOVA). **(E)** 10 μM of PF-543 or 5c apparently suppressed activity of SphK in GMCs by LC-MS/MS. Values represent means ± S.E. (*n* = 3 independent experiments.**P < 0.01 vs. DMSO by one-way ANOVA). **(F)** 10 μM of 5c suppressed protein level of pTyr255 CK2α in HG-induced GMCs, but 10 μM of PF-543 failed to do this. Values represent means ± S.E. (*n* = 3 independent experiments. **P < 0.01 vs. NG+DMSO; ^##^P < 0.01 vs. HG+DMSO by one-way ANOVA).

The aforementioned data indicated that CK2α was required for SphK1-mediated activation of NF-κB. To fully assess the regulatory function of SphK1 on CK2α activity in GMCs, overexpressions of wild-type SphK1 and SphK1 G82D were applied to study phosphorylation of CK2α at site of tyrosine 255, which was an indicator for CK2α kinase activity [33]. Data showed that protein level of pTyr255 CK2α was obviously upregulated after SphK1 overexpression in diabetic state or in non-diabetic state, whereas SphK1 G82D overexpression, had no effect on CK2α activation (Figure 9A). Furthermore, knockdown of SphK1 suppressed protein level of pTyr255 CK2α in HG-induced GMCs (Figure 9B).

PF-543, a competitive inhibitor to SphK1with an IC_50_ value of 3.6 nM and 5c, a non-competitive inhibitor to SphK1with an IC_50_ value of 3.3 μM, show inhibitory effect on enzyme activity of SphK1 [34–36]. MTT assay was applied to test cytotoxic effect of PF-543 and 5c in GMCs, suggesting that there were no evident inhibitory effect on cell growth when their concentrations were lower than 20 μM (Figure 9C & 9D). 10 μM of 5c suppressed activity of SphK1, followed by decrease of protein level of pTyr255 CK2α in HG-induced GMCs, whereas 10 μM of PF-543 which showed pharmacological inhibition on SphK1 like 5c, in contrast, failed to activate CK2α (Figure 9E & 9F).

### CK2α interacted with SphK1 in GMCs

**Figure 10:**
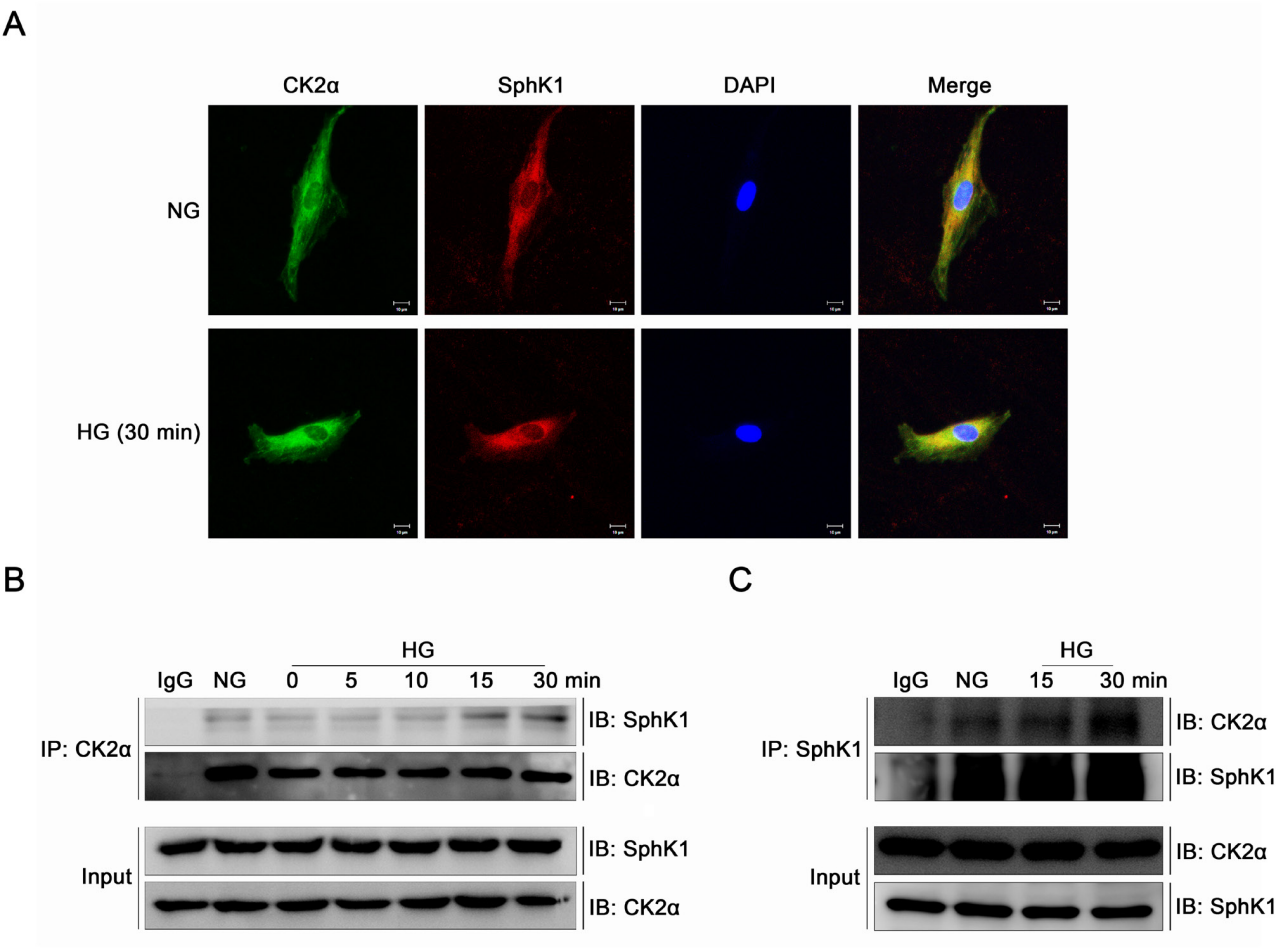
CK2α interacted with SphK1 in GMCs **(A)** It was indicated by immunofluorescence that CK2α and SphK1 showed visible cytoplasmic co-localization in GMCs, which strengthened apparently after 30 min of HG treatment. **(B** and **C)** Co-immunoprecipitation and reverse co-immunoprecipitation assay were applied to detect the interaction between CK2α with SphK1. This interaction reached a peak at 30 min after HG treatment.

Immunofluorescence and immunoprecipitation were performed to study the interaction of CK2α and SphK1 in GMCs further. It was indicated by immunofluorescence that CK2α and SphK1 showed visible cytoplasmic co-localization in GMCs which strengthened apparently after 30 min of HG treatment (Figure 10A). To further investigate the interaction between the two kinase, GMCs were lysed and total proteins were extracted for enrichment of CK2α or SphK1 with target antibodies respectively. As shown in the co-immunoprecipitation and reverse co-immunoprecipitation results, CK2α and SphK1 interacted with each other in NG condition and this interaction increased visibly after HG treatment in a time-dependent manner, reaching a peak value at 30 min (Figure 10B & 10C).

## DISCUSSION

Previous studies suggest that SphK1 appears to act as an oncogenic enzyme regulating various processes which is important in cancer progression and even shows physiological regulatory functions on antiapoptosis, transformation, proliferation, and survival of tumor cells [37–39]. Accumulating evidence suggests that SphK1 is closely associated with inflammation, cardiovascular system and immune system, affecting diabetic nephropathy, angiogenesis, vascular maturation, smooth muscle cell proliferation and immune inflammatory regulation [40–42].

Elevated activity and expression of SphK1 have been observed in the development and progression of DN [22]. Activation of SphK1 induced by HG stimulation promotes S1P formation to trigger phosphorylation of c-Jun and c-Fos, which upregulates expressions of FN and TGF-β1 and leads to diabetic renal fibrosis. AP-1, a dimer mainly formed by proteins of the Fos and Jun, is activated under HG stimulation and directly binds with its binding sites in SphK1 promoter to trigger SphK1 gene transcription, forming a positive feedback loop in diabetes [43]. In the current study, protein levels of SphK1 were significantly upregulated in kidneys of diabetic mice and rats. *In vitro*, SphK1 was activated and its protein level was significantly upregulated, accompanied by elevated expressions of FN and ICAM-1 in respond to HG stimulation. Furthermore, overexpression of exogenous SphK1 induced high expressions of FN and ICAM-1, while knockdown of SphK1 downregulated protein levels of FN and ICAM-1 in GMCs. *In vivo*, SphK1 has been considered to be a modulator on renal lesions, and FN is the major component of glomerular ECM, of which excessive generation performs as a dilapidator to provoke glomerular fibrotic lesions [44, 45]. We demonstrated that SphK1^-/-^ diabetic mice not only exhibited less generation of collagenous fibers and ECM, but also displayed less lesions and FN accumulation in glomeruli of kidneys than C57 diabetic mice.

In recent years, the correlation between SphK1 and DN has gradually become a hot topic, followed by an opinion that diabetic renal fibrotic disease is one kind of chronic inflammatory response in kidneys [5, 46]. SphK1 is also involved in the activation of several inflammatory responses during the regulation of cell physiological activities. Moreover, IL-1β, IF-γ and LPS induced inflammation via activating SphK1. Actually, process by which cyclooxygenase-2 (COX-2) induces inflammation under TNF-α treatment is inseparable from the activation of SphK1 [32, 47]. Notably, TNF-α stimulation activates SphK1 to increase expressions of VCAM-1 and ICAM-1, initiating formation of inflammatory infiltration around cells, which at last, leads to aggravate the situation of inflammation [48]. Data from Billich manifests that mRNA expression of SphK1 is upregulated in inflammation [49].

To further confirm the relationship between SphK1 and inflammation in the setting of DN, activation of NF-κB signaling pathway considered to be related to diabetic renal fibrosis was evaluated. As nuclear translocation of NF-κB is the main way of NF-κB-related regulation on gene transcription, we demonstrated that overexpression of exogenous wild-type SphK1 but not SphK1 G82D augmented nuclear translocation of NF-κB in GMCs. *in vivo*, NF-κB nuclear accumulation was upregulated in glomeruli of diabetic mice, but downregulated in SphK1^-/-^ diabetic mice versus C57 diabetic mice. The *in vivo* and *in vitro* experiments basically confirmed the regulatory effect of SphK1 on NF-κB-mediated diabetic renal fibrosis.

CK2α, a crucial kinase involved in inflammation and cell proliferation, mediates activation of NF-κB by interacting with IκBα in HG-treated GMCs, which is a novel and important way for regulation on DN [21]. For investigating the regulatory mechanism between SphK1 and CK2α, activation of NF-κB was measured in GMCs including NF-κB nuclear translocation, transcriptional activity and DNA binding activity after overexpression of exogenous CK2α while expression of SphK1 was knocked down by siRNA. As NF-κB was activated by exogenous CK2α overexpression, knockdown of SphK1 failed to block CK2α-induced NF-κB activation. By contrast, knockdown of CK2α markedly suppressed SphK1-induced NF-κB activation, suggesting that CK2α may be the downstream target protein of SphK1, and activation of NF-κB was mediated by SphK1 via CK2α during DN.

Results described in this study suggested that CK2α and SphK1 interacted with each other in NG condition and this combination was stronger after HG treatment in a time-dependent manner, which not only pointed out the close interaction between CK2α and SphK1 in the cytoplasm of GMCs, but also provided important evidence for regulation of SphK1 on CK2α activation. Overexpression of wild-type SphK1 but not SphK1 G82D activated CK2α in non-diabetic state, and activity of CK2α further increased in SphK1-overexpressed GMCs upon HG stimulation. Remarkably, increase of CK2α activity occurred in respond to HG treatment but declined after SphK1 knockdown. The above results indicated that activation of CK2α induced by SphK1 may be an essential mechanism in CK2α-mediated diabetic renal fibrosis.

Previous studies have shown that SphK1 mediates downstream signaling pathways by catalyzing the phosphorylation of sphingosine to S1P, whereas substrates that can be directly phosphorylated by SphK1 are rarely reported [50, 51]. Two selective inhibitors of SphK1 were performed to explore the phosphorylation of CK2α by SphK1. PF-543, a competitive inhibitor and 5c, a non-competitive inhibitor to SphK1 showed apparent inhibitory effect on enzyme activity of SphK1. As the phosphorylation level of CK2α at the tyrosine site of 255 reflects its kinase activity, data suggested that 5c, but not PF-543, inhibited activation of CK2α in HG-induced GMCs, suggesting that CK2α activation by SphK1 may not depend on generation of S1P. Unlike competition between PF-543 and sphingosine on binding sites of SphK1, 5c may inhibit phosphorylation and activation of CK2α by binding SphK1 to inhibit its overall activity, and the process may not depend on the presence of S1P.

Taken together, the present study *in vitro* and *in vivo* demonstrated that SphK1 mediated pathological process of diabetic renal fibrosis via NF-κB pathway. SphK1 may be an upstream kinase of CK2α and even phosphorylated CK2α to activate downstream NF-κB pathway. Further investigation will be needed to explore the detailed relationship between CK2α and SphK1.

## MATERIALS AND METHODS

### Reagents and antibodies

Low glucose-Dulbecco’s Modified Eagle Medium (DMEM), fetal bovine serum (FBS), RNAiMAX transfection reagent, LTX reagent with PLUS TM reagent and EMSA kit were purchased from Life Technologies (Grand Island, NY, USA). D-Glucose, antibody against pTyr255 CK2α, streptozotocin (STZ), thiazolyl blue tetrazolium bromide (MTT), dimethyl sulphoxide (DMSO) and ATP were obtained from Sigma–Aldrich Corporation (St. Louis, MO, USA). Antibodies against NF-κB p65, CK2α, Tubulin, Lamin B1 and SphK1 (antibody against SphK1 was used for western blotting, immunofluorescence and immunohistochemistry) were produced by Abcam (Cambridge, MA, USA). Antibodies against FN, ICAM-1, IκBα and SphK1 (antibody against SphK1 was only used for immunoprecipitation) were from Santa Cruz Biotechnology (Dallas, TX, USA). Flag-tagged human wild-type SphK1 and the dominant negative SphK1 (SphK1 G82D) were kindly provided by Dr. Pu Xia (Australia). Horseradish peroxidase-conjugated secondary antibodies and Dual-Glo^®^ Luciferase Assay System were obtained from Promega Corporation (Madison, WI, USA). Alexa Fluor^®^ 488 goat anti-mouse IgG, Alexa Fluor^®^ 488 goat anti-rabbit IgG and Alexa Fluor^®^ 594 donkey anti-goat IgG were purchased from Thermo Fisher Scientific (Rockford, IL, USA). Nuclear extract kit was purchased from Active Motif (Carlsbad, CA, USA). The sequences on small-interfering RNA of SphK1 or CK2α were from Gene Pharma (Shanghai, China). SphK1 inhibitor 5c and S1P were obtained from Cayman Chemical Company (Ann Arbor, Michigan, USA). SphK1 inhibitor PF-543 was obtained from Selleck. C17-sph was purchased from Avanti Polar Lipids (Alabaster, AL, USA).

### Animal experiments

All animals except SphK1^-/-^ mice (kindly provided by Richard L. Proia, NIH) were purchased from the Laboratory Animal Center, Sun Yat-sen University, Guangzhou, China, and housed under specific pathogen-free conditions. Procedures for animals treatment were implemented in accord with the China Animal Welfare Legislation and were reviewed and approved by the Sun Yat-sen University Committee on Ethics in the Care and Use of Laboratory. The mice and rats were adapted to the environments for one week before treatment. Fasting blood glucose (FBG) levels were detected, and mice with a value more than 11.1 mM or rats with a value more than 16.7 mM were regarded as diabetic models.

Animal Quality Certificate Number of male C57/BL6 mice (n = 10, 20 ± 2 g), db/db mice (n = 10, 20 ± 2 g) and KKAy mice (n = 10, 20 ± 2 g) was 2014032122. Experimental mice were divided into three groups as follows: C57 mice group (n = 8), db/db mice group (n = 8), KKAy mice group (n = 8).

Animal Quality Certificate Number of male C57/BL6 mice (n = 20, 20 ± 2 g) was 20163000562. One half of the mice were induced by intraperitoneal injection of freshly prepared STZ (40 mg/kg) in citrate buffer once a day for five consecutive days. Other ten of the mice were injected with an equal volume of citrate buffer. FBG levels of STZ-induced mice were measured one week after STZ injection, and mice were divided into two groups as follows: C57 control group (n = 8), diabetic mice group (n = 8).

Animal Quality Certificate Number of male SD rats (n = 20, 180 ± 10 g) was 20164000744. One half of the rats were induced by one single intraperitoneal injection of freshly prepared STZ (40mg/kg) in citrate. Other ten of the rats were injected with an equal volume of citrate buffer. FBG levels of STZ-induced rats were measured one week after STZ injection, and rats were divided into two groups as follows: SD control group (n = 8), diabetic rats group (n = 8).

Animal Quality Certificate Number of male C57/BL6 mice (n = 20, 20 ± 2 g) and SphK1^-/-^ mice (n = 20, 20 ± 2 g) was 2014035372. One half of the C57 mice and SphK1^-/-^ mice were induced by intraperitoneal injection of freshly prepared STZ (40 mg/kg) in citrate buffer once a day for five consecutive days. Others were injected with an equal volume of citrate buffer. FBG levels of STZ-induced C57 mice and SphK1^-/-^ mice were measured one week after STZ injection, and mice were divided into four groups as follows: C57 control group (n = 8), C57 diabetic mice group (n = 8), SphK1^-/-^ control group (n = 8), SphK1 ^-/-^ diabetic mice group (n = 8).

Experimental animals were feed for eight weeks and were sacrificed, and kidney samples were collected for detection.

### Cell culture

Primary GMCs were separated from Sprague-Dawley (SD) rats (around 150 g) and identified via a specific assay as previously describe [25]. Cortex of kidneys were cut into pieces in ice-cold PBS, and the lumps were harvested to filtrate through three specific meshes orderly (sizes of meshes were 175, 147 and 74 μm). Ultimately, the matter upon 74 μm mesh was collected, seeded in culture bottle and incubated in 10% FBS DMEM at 37°C with 5% CO_2_ after digestion with 0.1% collagenase IV for 15-25 min. Passages of GMCs from 5th to 12th were used for experiments, and GMCs were rendered quiescent by incubation in serum-free medium for 24 h before treating with glucose or other stimuli while 80% confluent.

### Western blot assay

Western blot assay was performed with the standard protocol as previously described [52]. Cultured GMCs or kidney tissues were harvested and lysed for extracting proteins. RIPA with both protease and phosphatase cocktail was applied to extract total proteins, and nuclear extract kit was performed to extract nuclear and cytoplasmic proteins. Proteins from GMCs or tissues were separated by 8% sodium dodecyl sulfate-polyacrylamide gel electrophoresis (SDS-PAGE), and transferred to PVDF membrane for blocking with defatted milk at room temperature. Membranes with the target proteins were incubated at 4°C with primary antibodies overnight. After incubation exceed 12 h, membranes were washed with 0.1% Tween-20/TBS for three times in 30 min and incubated with the second antibodies at room temperature for 1 h. Signals were recorded by ImageQuant LAS4000mini produced by GE healthcare (Waukesha, WI, USA) and analyzed by the Quantity One Protein Analysis Software produced from Bio-Rad Laboratories (Hercules, CA, USA).

### Immunofluorescence

GMCs were seeded and cultured on glass coverslips for 24 h before treatment. After washing with ice-cold PBS for three times, GMCs were fixed with 4% paraformaldehyde for 15 min and then permeabilized with 0.1% Triton X-100 for 10 min at room temperature. After another washing with ice-cold PBS for three times, GMCs were blocked with 10% goat serum for 30 min and then incubated with primary antibodies overnight at 4°C. After incubation exceed 12 h, GMCs were washed again and incubated with fluorescent secondary antibody for 1 h away from light at room temperature. 4',6-diamidino-2-phenylindole (DAPI, Sigma, USA) was applied to stain nucleus for 10 min. Fluorescent signals were recorded by a laser scanning confocal microscope (LSM710, Carl Zeiss, Germany).

### SphK activity assay

SphK activity was measured via a specific assay as previously described. 2.5 μL of 200 μM C17-Sph dissolved in 5% Triton X-100 and 2.5 μL of 20 mM ATP containing 200 mM MgCl_2_ were mixtured with 30 μg proteins to a final volume of 50 μL. After incubation at 37°C for 20 min, the reaction was terminated by adding with 5 μL of 1 M HCl and 200 μL of mix consisted of chloroform, methanol and HCl (100: 200: 1, v/v). 10 μL of 1 μg/mL S1P, an internal standard for LC-MS/MS detection, was added into the reaction solution, and then 60 μL of chloroform and 60 μL of 2 M KCl were added. Oil and water phase were generated after centrifugation at 12,000 g for 5 min at 4 °C, and then the oil phase was transferred into a 1.5 mL tube to vacuum-dry for 60 min. The residue was dissolved with mix consisted of methanol: 0.1% formic acid (95:5), and 10 μL of solution was detected by LC-MS/MS system (Thermo Finnigan, Silicon Valley, CA, USA).

### Transfections of plasmids and small-interfering RNA

Transfections of plasmids including Flag-tagged wild-type SphK1 and SphK1 G82D were applied to GMCs according to the manufacturer’s instruction for LTX reagent with PLUS™ reagent. GMCs were cultured with 10% FBS DMEM for 24 h prior to transfection by which 2 μg of plasmids was transfected into cells and then incubated for 48 h to harvest. Oligonucleotides targeting CK2α or SphK1 for siRNA were produced from Gene Pharma, and the effective sequences were respectively as follows:

CK2α forward: 5’-GCCΑUCΑΑUΑUCΑCΑΑΑUΑTT-3’;
CK2α reverse: 5’-UΑUUUGUGΑUΑUUGΑUGGCTT-3’;
SphK1 forward: 5’-GACGGCAACUCUAUUCUGUTT-3’;
SphK1 reverse: 5’-ACAGAAUAGAGUUGCCGUCTT-3’.

The sequences for siRNA were transfected using RNAiMAX transfection reagent according to the manufacturer’s protocol and then incubated with cells for 48 h.

### Dual luciferase reporter assay

GMCs were seeded and cultured in 96-well plates with 10% FBS DMEM for 24 h prior to treatment. 0.2 μg pNF-κB-Luc (Beyotime, Haimen, China) and 0.04 μg pRL-TK (Promega) were co-transfected into GMCs using LTX reagent with PLUS™ reagent for 48 h according to the manufacturer’s instructions. Finally, cells were harvested and lysed, and luciferase activity of firefly or renilla were assessed according to Dual-Glo^®^ Luciferase Assay System. Signals were normalized to the renilla luciferase activity.

### Electrophoretic mobility shift assay (EMSA)

EMSA was performed to measure the DNA binding activity of NF-κB according to manufacturer’s instruction. 5 μg of nuclear proteins was incubated with the cocktail consisted of 50 ng/ml poly (dI-dC), 0.05% Nonidet P-40, 5 mM MgCl_2_ and 2.5% glycerol for 10 min, and then incubated with NF-κB probes for additional 20 min at room temperature. The samples were transferred to nylon membrane for DNA-protein crosslinks after separation by 7% nondenaturing PAGE. Membranes were blocked with blocking buffer for 1 h and incubated with horseradish peroxidase-conjugated streptavidin antibodies (1:300) for another 15 min. Signals were visualized with enhanced chemiluminescence and recorded by ImageQuant LAS4000mini.

### MTT assay

The MTT assay was used in measurement of cell proliferation according to protocol as previously described [52]. GMCs were seeded and cultured in 96-well plates with 10% FBS DMEM for 24 h prior to treatment. Different concentrations of PF-543 or 5c were used to treat GMCs cultured in NG or HG condition in a dose-dependent manner. 0.5 mg/mL of MTT was added to each well and incubated with cells for 4 h prior to harvest. Medium of each well was abandoned, and then 100 μL DMSO was added to dissolve crystal solid. The absorbance was detected and recorded at the wavelength of 570 nm by a microplate reader from Bio-Tek (Winooski, VT, USA).

### Immunoprecipitation

GMCs were harvested and lysed with immunoprecipitation buffer on ice for 30 min. Supernatant was collected after centrifuging at 12,000 g for 10 min at 4°C, and 500 μg of proteins was incubated with 2 μg of antibodies against target protein or IgG overnight at 4°C keeping shaking. 30 μL of agarose A/G beads was added and incubated with the mixture for 4 h at 4°C. After incubation, samples were washed three times with washing buffer and 25 μL of SDS loading buffer was added to the bead-protein conjugate to mix. In the end, samples were subjected to western blot assay with respective antibodies.

### Statistical analysis

All experiments were performed at least three times with similar results. Data were analyzed by Unpaired Student’s t test for comparison between two groups, and by one-way ANOVA with post hoc multiple comparisons for multiple comparisons. P < 0.05 was considered statistically significant.

## Abbreviations

SphK1: sphingosine kinase 1
CK2α: casein kinase 2α subunit
DN: diabetic nephropathy
DM: diabetes mellitus
ECM: extracellular matrix
FN: fibronectin
ICAM-1: intercellular adhesion molecule-1
NF-κB: nuclear factor of kappa B
IκB: inhibitor of kappa B
IKK: inhibitor of kappa B kinase
GMCs: glomerular mesangial cells
NG: normal glucose
HG: high glucose
AGEs: advanced glycation end products
S1P: sphingosine-1-phosphate
IL-1β: interleukin1β
IF-γ: interferon γ
LPS: lipopolysaccharide
TNF-α: tumor necrosis factor-α
DMEM: Dulbecco’s Modified Eagle Medium
FBS: fetal bovine serum
MTT: thiazolyl blue tetrazolium bromide
DMSO: dimethyl sulphoxide
siRNA: small-interfering RNA
WB: western blotting
EMSA: electrophoretic mobility shift assay
LC-MS/MS: liquid chromatography tandem-mass spectrometry
FBG: fasting blood glucose
SD: Sprague-Dawley
STZ: streptozotocin
PAS: periodic acid-schiff
HE: hematoxylin-eosin
